# Square-stepping exercise in older inpatients in early geriatric rehabilitation. A randomized controlled pilot study

**DOI:** 10.1186/s12877-024-04932-3

**Published:** 2024-04-10

**Authors:** Katja Fränzel, Jessica Koschate, Ellen Freiberger, Ryosuke Shigematsu, Tania Zieschang, Svenja Tietgen

**Affiliations:** 1 Department of Geriatrics, Carl von Ossietzky Universität Oldenburg Faculty VI Medicine and Health Sciences, Department of Health Services Research, Ammerländer Heerstraße 140, Oldenburg, 26129 Germany; 2 Institute for Biomedicine of Ageing Friedrich-Alexander-University Erlangen-Nürnberg (FAU), Kobergerstr. 60, 90408 Nuremberg, Germany; 3 School of Health and Sport Science, Chukyo University, 101 Tokodachi, Toyota, Aichi 470-0393 Japan; 4 Department of Geriatrics, General Hospital Bremerhaven Reinkenheide gGmbH, Postbrookstraße 103, 27574 Bremerhaven, Germany

**Keywords:** Square-Stepping Exercise, Physical function, Early geriatric rehabilitation, Gait characteristics

## Abstract

**Background:**

Preservation of mobility and fall prevention have a high priority in geriatric rehabilitation. Square-Stepping Exercise (SSE) as an evaluated and standardized program has been proven to be an effective training for older people in the community setting to reduce falls and improve subjectively perceived health status. This randomized controlled trial (RCT), for the first time, examines SSE in the context of inpatient early geriatric rehabilitation compared to conventional physiotherapy (cPT).

**Methods:**

Data were collected in a general hospital in the department of acute geriatric care at admission and discharge. Fifty-eight inpatients were randomized to control (CG, *n* = 29) or intervention groups (IG, *n* = 29). CG received usual care with cPT five days per week during their hospital stay. For the IG SSE replaced cPT for at least six sessions, alternating with cPT. Physical function was measured with the Short Physical Performance Battery (SPPB) and Timed “Up & Go” (TUG). Gait speed was measured over a distance of 10 m. In a subgroup (*n* = 17) spatiotemporal gait parameters were analyzed via a GAITRite® system.

**Results:**

Both the SPPB total score improved significantly (*p* =  < 0.001) from baseline to discharge in both groups, as did the TUG (*p* < 0.001). In the SPPB Chair Rise both groups improved with a significant group difference in favor of the IG (*p* = 0.031). For both groups gait characteristics improved: Gait speed (*p* =  < 0.001), walk ratio (*p* = 0.011), step length (*p* =  < 0.001), stride length (*p* =  < 0.001) and double support (*p* = 0.009). For step length at maximum gait speed (*p* = 0.054) and stride length at maximum gait speed (*p* = 0.060) a trend in favor of the IG was visible.

**Conclusions:**

SSE in combination with a reduced number of sessions of cPT is as effective as cPT for inpatients in early geriatric rehabilitation to increase physical function and gait characteristics. In the Chair Rise test SSE appears to be superior. These results highlight that SSE is effective, and may serve as an additional component for cPT for older adults requiring geriatric acute care.

**Trial Registration:**

DRKS00026191.

**Supplementary Information:**

The online version contains supplementary material available at 10.1186/s12877-024-04932-3.

## Background

Rehabilitation in older people is known to be an important component in maintaining or regaining independent living after an acute event such as a stroke, hip fracture, etc. Geriatric rehabilitation itself is defined as an approach of multidimensional diagnostic and therapeutic interventions for patients affected by multimorbidity and geriatric syndromes. The aim is to focus on individual preferences and deficits, maintain functional reserve and improve physical function. Across Europe, the structure of geriatric rehabilitation varies [1]. In Germany, geriatric rehabilitation is provided post-acutely in rehabilitation centers or during hospitalization for acute illness, called early geriatric rehabilitation. Early geriatric rehabilitation is aimed at geriatric inpatients with acute illnesses (e.g. stroke, pneumonia, hip fracture). The length of stay is usually two to three weeks. The core principle is a treatment by a multiprofessional team with daily physiotherapy, occupational therapy, speech therapy, and neuropsychological therapy. The main goals are to maintain independence and regain the ability to perform activities of daily living. These two components are included under the umbrella term of mobility, which is closely related to physical and mental health in older age [2, 3]. This also corresponds to the concept of healthy ageing as defined by the World Health Organization (WHO) [4]. Although physiotherapy plays a key role in mobilization in this setting there are no clear recommendations, and evidence for early rehabilitation in geriatric patients in Europe is currently inconclusive. As a result, different reviews on the benefits of early rehabilitation in hospitalized older people show heterogeneous results [5–7]. In the included studies, the intervention content of the rehabilitation treatment varies. In general, it is not described which specific interventions were chosen as part of the physiotherapy treatment. There is also little information about the duration, intensity and frequency of the chosen interventions [6, 8]. Furthermore, the choice of outcome measurements varies, leading to heterogeneous results across studies [7]. The individuality of the interventions used additionally complicates standardization. Therefore, further research with standardized programs in this specific setting is needed. The introduction of specific and well-studied exercise programs to improve lower limb function is particularly important for maintaining mobility and preventing falls. An evaluated and effective intervention for older people in the community setting is the Square-Stepping Exercise (SSE) program [9, 10]. SSE combines a specific and adaptive approach. It is a simultaneous motor-cognitive training that focuses on the lower limb function for motor skills and on executive function and working memory for cognitive skills. By combining a physical and a cognitive aspect, SSE seems to be particularly suitable as exercise intervention in the geriatric clientele. It is also easy to implement, inexpensive, and offers a playful additional component for conventional physiotherapy. Originally developed by Shigematsu and Okura in Japan, the program was primarily designed to improve the functional fitness of the lower extremities. The aim was to improve the walking ability of older people in the general population and to minimize the risk of falls [9, 10]. In SSE, step patterns are performed on a mat consisting of 40 squares. The level of difficulty is adaptive and can be supplemented with additional elements. SSE follows the principle of proactive and reactive response improvement. This improves the ability to regain balance by taking a corrective step after a stumble. SSE training has been shown to be effective in improving physical function [9–11]. In addition, due to the high cognitive demands, SSE in older people has been shown to have an additional effect on cognitive performance [12] in the functional domains of executive function and working memory. Fisseha et al. [13] provided an overview of the study landscape in their systematic review and meta-analysis of the effectiveness of SSE for fall prevention and fall injuries. SSE was found to be effective in reducing fear of falling in older people, as well as improving their subjectively perceived health status.

To date, SSE has only been studied in older people living independently. Earlier studies investigated the effects of SSE over a training period of several months [12, 14–18]. Currently, two studies investigated the effects of SSE in shorter training periods. Gan et al. [19] compared a home-based online trial to in-person SSE training and showed short term effects of SSE on cognitive and social functions after 3 weeks of training in both groups. Similar result was obtained by Harshika et al. comparing a 4-week SSE program with a balance training program [20]. Ravichandran et al. [21] confirmed that a 4-week SSE program can effectively improve gait and balance in older adults with Parkinson's disease. SSE proved to be superior to cPT in these two parameters. These aspects prompted us to investigate this exercise program in the context of inpatient early geriatric rehabilitation. The average duration of intervention in this setting is three weeks.

The aim of the present pilot study was to evaluate the effect of SSE on physical functioning and gait characteristics compared with conventional physiotherapy (cPT). A further aim was to assess the effect of SSE on executive functions due to the high cognitive requirement. As this is the first trial of SSE training in early geriatric rehabilitation with the accompanying time restriction, feasibility (acceptance and safety) was also an objective. Acceptance was quantified by potential dropouts due to non-adherence. Safety was quantified by observable falls during training.

## Methods

### Study design

This study was designed as a single-blinded, randomized, controlled intervention trial. It was conducted in a general hospital in the department of acute geriatric care in Bremerhaven, Germany in accordance with the Declaration of Helsinki. All procedures were approved by the Ethics Committee of the Bremen Medical Association. It was registered at https://www.drks.de/ on 18/10/2021 (DRKS00026191). All procedures were carried out with written informed consent of the participants. The results presented here focus on mobility, which is composed of physical function and gait characteristics. These outcomes were quantified using the Short Physical Performance Battery (SPPB), the Timed "Up and Go" (TUG)-test and gait speed (semi-objective via stopwatch). In a subsample of 17 participants effects on gait characteristics were measured by computerized spatial and temporal gait measurement (GAITRite® system) under laboratory conditions. At the beginning of the study, this measurement tool was not yet available, which is why only the most recently recruited participants were included. The study was planned for a duration of 18 months. The actual study period until the planned sample size was reached was from November 2021 to August 2022.

### Study population

The sample size calculation was based on the results for change in the Short Physical Performance Battery (SPPB) by Sebastião et al. [16]. On average, the score for the SPPB changed from 8.8 ± 2.6 to 9.5 ± 2.1 points in the intervention groups and from 7.2 ± 3.3 to 7.1 ± 4 points in the control group (η2 = 0.055). The function "F-test, ANOVA: repeated measures, within-between interaction" of the software G*Power 3.1.9.4 was used. Assuming an α error probability of 5% and a power (1-β) of 95%, as well as two groups and two measurement times and η2 = 0.055, a required total sample size of *N* = 58 resulted.

During the survey period from November 2021 to August 2022 patients were screened for eligibility on admission to the department of acute geriatric care. Study inclusion criteria included participation in inpatient early geriatric rehabilitation, ability to walk short distances (10 m) in company without walking aid, Mini Mental State Examination (MMSE) score > 22 to ensure ability to consent, sufficient cognitive skills to implement the training program and sufficient knowledge of German or English. Excluded were patients with aphasia to an extent that participation in the study is not possible, severe visual limitations, high grade presbyacusis, severe impairment of physical functionality, and limitations of functions of the arms and legs with the inability to walk. Subjects meeting the inclusion criteria were randomized to intervention group (IG) or control group (CG) before baseline testing. Via block randomization with permuted blocks of variable length eligible inpatients (*N* = 72) were randomized to the CG (*n* = 36) or the IG (*n* = 36). A digital, password-protected list of identification numbers was created via this randomization. The list of allocation order was kept by a third uninvolved person. The participants were randomized according to the order of this list. Data were collected before the start of the intervention and before discharge from hospital. Recruitment was completed when a complete data set of 58 participants was reached. If participants dropped out or were excluded, recruitment continued until the sample size was complete.

### Measurements

Measurements were taken after randomization and at the end of the intervention by trained staff using standardized tests. The investigator was blinded to the randomization.

#### Physical function assessment

Short Physical Performance Battery [22] consists of 3 tests: a test for balance (SPPB Balance), a test for walking over 4 m (SPPB Speed) and a test for standing up from a chair five times (SPPB Chair Rise). The time interval required to complete the test is converted into scores (0–12). The maximum number of points per test is 4. Higher scores indicate higher levels of functioning.

Timed “Up & Go” [23]: The time is measured with a stopwatch in which a seated patient stands up, walks 3 m at habitual speed, turns around, walks back and sits down again.

#### Gait assessments

##### Gait Speed

Gait speed was measured manually with a stopwatch on a distance of 10 m with a marked start and stop line. This also included the acceleration phase. Gait analysis was performed according to a standardized protocol. The patients completed two walks at habitual speed, two at maximum walking speed to measure reserve capacity, and one walk under dual task conditions to measure motor cognitive interference. The dual task was freely selectable from the variants enumerate animals, enumerate plants, subtract in the number range 0–100 (100 minus 3 or 100 minus 7). The variants had to differ from each other in the pre- and post-measurement in order to avoid repetition effects. In the motor-cognitive test condition no prioritization information was given to the participants.

##### Spatiotemporal Gait Parameters

In order to be able to record spatiotemporal gait parameters as well, an electronic gait analysis was performed in a subgroup in addition to the manual recording of gait speed. In accordance with the current gold standard for the analysis of temporal and spatial gait parameters, the analysis of gait parameters was performed using the GAITRite® system. An 8-m electronic gait mat equipped with measurement sensors was used (6,10 m walkway, 700 cm long, active electronic surface area 61 cm × 610 cm, total 23,040 pressure sensors, sample rate 120 Hz, GAITRite®, CIR Systems Inc., Frankling, USA). The validity and reliability of this measurement system has been confirmed in several studies [24, 25]. The gait analysis was performed in compliance with the guidelines for objective gait analysis (e.g., room conditions, footwear) of the Biomathics and Canadian Gait Consortium Initiative [26].

There are different frameworks that characterize gait and from which gait parameters to be measured can be derived [27–29]. In these frameworks, gait parameters relate to different variables: Data-related, associated with cognitive decline, and disease-related. Based on this literature, Dapp and colleagues derived references for gait analysis by extracting overlapping gait parameters. This was a geriatric clientele, which also includes patients with frailty. Ultimately, five parameters were extracted that were determinants of functional level [30].

To the authors' knowledge, the review of Dapp and colleagues is the most recent study, which is why we used the same parameters in this study:

- Gait velocity (cm/s): Ratio of distance walked in centimeters divided by time elapsed in seconds.
- Stride length (cm): Distance in centimeters between the heel points of two consecutive footprints of the same foot on the line of progression.
- Walk ratio (cm/(steps/min)): ratio of step length in centimeters by step frequency in steps per minute.
- Double support time (ms): Period in milliseconds during gate cycle when both feet touch the ground at the same time.
- Stride length variability (%): Variability of stride length expressed as coefficient of variance (CV) in percent applying the formula: (standard deviation/mean)*100.

The gait analysis was performed according to the same standardized protocol as the manual measurement of gait speed.

#### Further assessments

##### Concern about falling assessment

To measure concerns about falls, the German version of the Falls Efficacy Scale-International (FES-I) was used in the 16-item version [31]. This questionnaire includes concerns about falls during light and heavy physical activities and during social activities. Concerns about falls in each activity are recorded on a 4-point scale (1 = not at all concerned, 4 = very concerned).

##### Assessment of health-related quality of life.

The EuroQol- 5 Dimension (EQ-5D) was used to assess health-related quality of life [32]. The EQ-5D measures the five dimensions mobility, self-care, usual activity, pain/discomfort, and anxiety/depression. The questionnaire also includes a Visual Analog Scale, which allows patients to rate their perceived health status on a scale from 0 (the worst possible health status) to 100 (the best possible health status).

### Clinical characteristics

Demographic data and functional status (Barthel Index at admission and discharge) were obtained from the patient's medical record [33]. Additional data, such as years of education, walking aids, and average walking distance, were obtained in a standardized interview.

### Intervention

To ensure comparability, both groups received the same number of training sessions of similar total duration during the three-week intervention period.

#### Control condition

The CG received standard of care with five 30-min sessions of physiotherapy a week. This corresponds to the usual therapy plan in geriatric rehabilitation, which provides for therapies only on weekdays.

#### Intervention condition

**Fig. 1:**
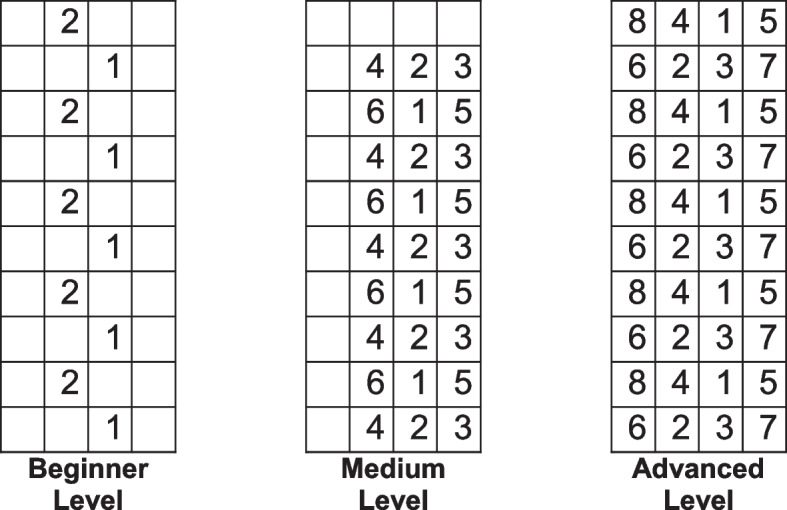
Examples of the step patterns in beginner, medium and advanced level

In the IG three of the five sessions were replaced by a 30-min session of SSE. Participants received standard care and SSE in alternation. This was carried out over the entire period of early geriatric rehabilitation. Therefore, the number of units may varied depending on the length of stay. But the ratio, usual physiotherapy/ SSE units, remained the same. The required minimum number of SSE units was six. In SSE training, a progressive training load was used according to a structured training plan. At the beginning of the session, the participants were familiarized with the carpet and learned how to walk across it. In the following, step patterns from the basic level were learned and increased in complexity according to the individual abilities. Level of difficulty was increased progressively. The physiotherapists assessed when the participant was sufficiently confident in a level to increase the complexity of the patterns. The step patterns and classification into requirement levels were taken from the original Shigematsu study [9, 11] and a selection of patterns to be used was made within our research group. After familiarization with the training program and confident learning of a pattern, a dual task was added. The participants were asked by the physiotherapists to complete an additional cognitive task, while walking the learned pattern. The physiotherapists were free to choose from predefined tasks (enumerating animals or plants, solving arithmetic tasks, e.g. 100 minus 6, alternating enumerating numbers and letters, verbal distraction). All physiotherapists involved in this pilot study were trained in the SSE program. Figure 1 shows examples of the step patterns of the different levels of difficulty.

### Statistical analysis

Unpaired t-tests and Chi-square-tests were used to compare demographic and clinical characteristics of CG and IG at baseline. To compare test performances of the two groups for the two time points (baseline = t1 and post intervention = t2) two-factor (group*time) ANOVA for repeated measures was used. In case of group comparisons of more than two groups, post-hoc tests with Bonferroni correction were applied. To compare test performance in the subtests of the SPPB, the difference in scores between t1 and t2 was first calculated. To analyze the frequencies, these were evaluated in a cross-tabulation according to Pearson Chi-Square. For all calculations, a significance level at α = 0.05 was used. All analyses were performed using SPSS version 29.0 (SPSS, Inc., Chicago, IL, USA).

## Results

**Fig. 2:**
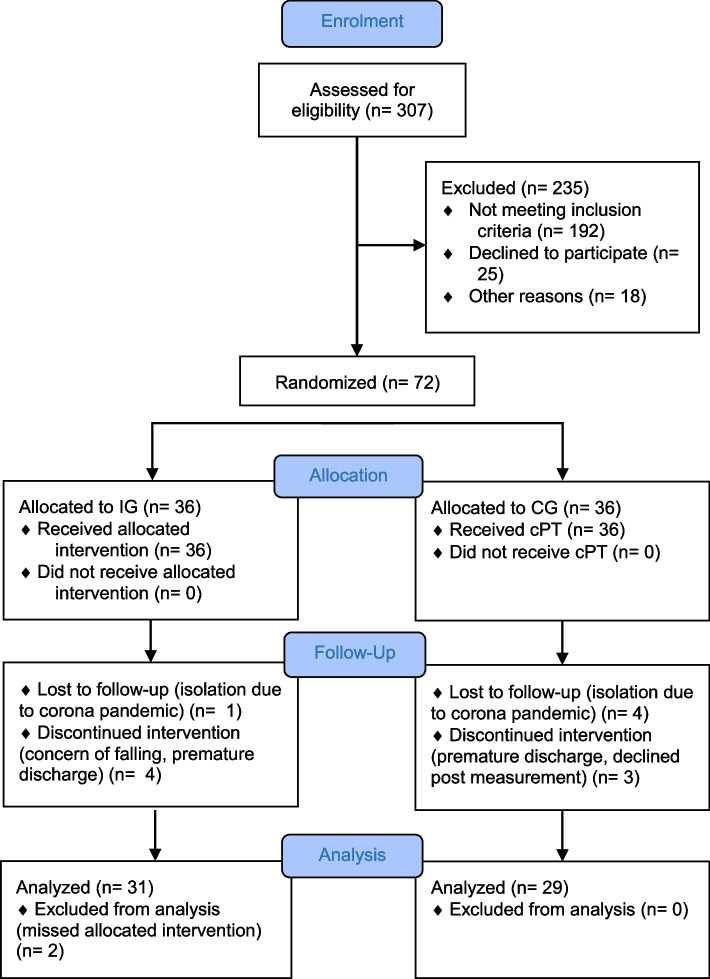
Participant flow chart Abbreviations: IG, intervention group; CG, control group; cPT, conventional physiotherapy

During the survey period, 307 patients were screened for eligibility on admission to the department of acute geriatric care and 115 patients met the inclusion criteria. Of these, 57 patients were excluded due to refusal to participate or other reasons. These included staff shortage and isolation of patients due to the Corona pandemic. In the CG there were 4 losts to follow-up (due to the Covid-19 pandemic) and 3 dropouts (due to Covid-19 pandemic or missing data) during the course of the study. In the IG there were 1 lost to follow-up (due to Covid-19 pandemic) and 4 dropouts (due to missing data, concern about falling or prematurely discharge from hospital). A total of 29 patients were included in the statistical analysis of the CG. A total of 31 patients were included in the statistical analysis of IG. Two patients were excluded from the analysis because they were not offered enough SSE units. The process of screening, enrolment, allocation, follow-up, and data analysis is shown in the Fig. 2.

The total number of physiotherapy units received during study participation was 11.72 ± 1.9 in CG and 12.07 ± 1.9 in IG (*p* = 0.492). In the IG, the total number of physiotherapy units included 6.62 ± 0.8 SSE units.

The participant´s mean age was 79.1 ± 6.9 years, the mean Barthel Index was 63.19 ± 12.66 points and the mean MMSE score was 27.7 ± 1.8 points. The baseline physical performance averaged 16.39 ± 10.36 s in the TUG, 6.83 ± 2.60 points in the SPPB and a mean maximum gait speed of 1.14 ± 0.32 m per second. No differences were found between the CG and IG at baseline for any variable (Table 1).

**Table 1:** Demographic and clinical characteristics of patients at baseline

**Characteristic**	**CG** (*n* = 29)	**IG** (*n* = 29)	***p*** **-value**
Age Mean ± SD, (min–max)	78.9 ± 6.73 (65–88)	79.4 ± 7.08 (63–90)	0.791
Woman (number)	13	14	0.792
Education (years) Mean ± SD, (min–max)	11.5 ± 1.97 (7–16)	12.2 ± 3.10 (7–20)	0.316
Mini mental status examination (score) Mean ± SD, (min–max)	27.9 ± 1.63 (23–30)	27.5 ± 1.95 (23–30)	0.386
Barthel Index (score) Mean ± SD, (min–max)	61.9 ± 12.06 (40–85)	64.5 ± 13.32 (40–90)	0.441
SPPB (score) Mean ± SD, (min–max)	7.03 ± 2.93 (1–12)	6.62 ± 2.24 (4–11)	0.549
TUG (sec) Mean ± SD, (min–max)	14.64 ± 6.65 (5–32)	18.07 ± 12.88 (7–78)	0.215
Gait speed (m/sec) Mean ± SD, (min–max)	0.93 ± 0.26 (0.5–1.43)	0.87 ± 0.20 (0.38–1.25)	0.315
Maximum gait speed (m/sec) Mean ± SD, (min–max)	1.19 ± 0.35 (0.73–1.85)	1.09 ± 0.27 (0.56–1.64)	0.261
FES-I (score) Mean ± SD, (min–max)	23.7 ± 6.98 (16–41)	23.3 ± 7.54 (16–52)	0.857
EQ-5D Level of health (%) Mean ± SD, (min–max)	62.5 ± 20.82 (20–100)	60.2 ± 21.19 (20–90)	0.686

*Abbreviations*: *SD* standard deviation, *CG* control group, *IG* intervention group, *SPPB* Short Physical Performance Battery, *TUG* Timed “Up & Go”, *FESI-I* Falls Efficacy Scale- International, *EQ-5D* EuroQol-5 Dimensions

The intervention was performed safely. There were no fall events or other adverse events.

### Effects of the intervention on physical functioning parameters

**Fig. 3:**
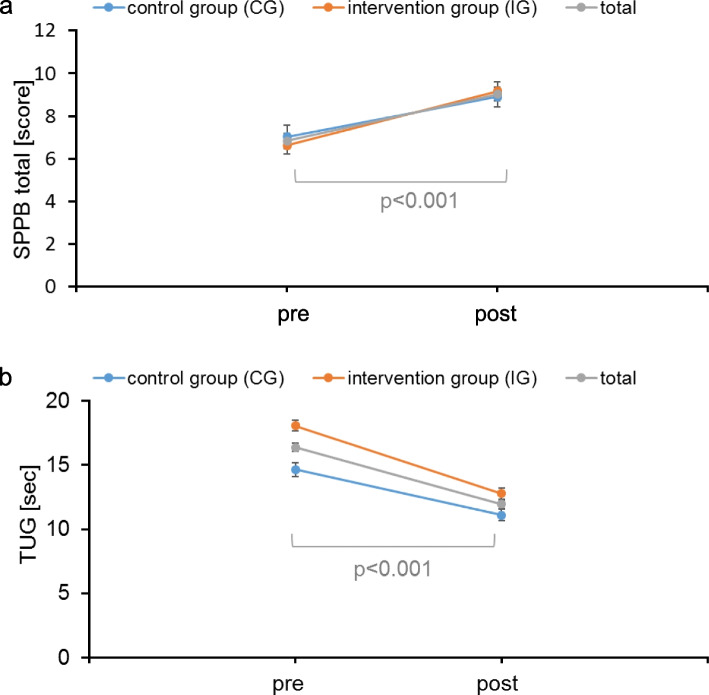
**a** Outcome measure SPPB total score. **b** Outcome measure TUG sec. Abbreviations: CG, control group IG, intervention group SPPB, Short Physical Performance Battery TUG, Timed “Up & Go”

Both groups improved functional performance in the SPPB and in the TUG from admission to discharge. Interactions of group allocation by time showed no statistically significant difference. The comparisons of the outcome measures are presented in Fig. 3 and Table 2.

**Table 2:** Effects of the intervention on SPPB total score TUG and gait variables

Physical function variables	Baseline CG: *n* = 29 IG: *n* = 29	Post intervention CG: *n* = 29 IG: *n* = 29	Time		Time*Group allocation		Group allocation	
			*p*-value	η^2^	*p*-value	η^2^	*p*-value	η^2^
**SPPB total (score)**	CG: 7.03 ± 2.93 IG: 6.62 ± 2.24	CG: 8.90 ± 2.51 IG: 9.17 ± 2.39	< 0.001	0.520	0.229	0.026	0.909	< 0.001
**TUG (sec)** **CG** **:** ***n*** **= 28**	CG: 14.64 ± 6.65 IG: 18.07 ± 12.88	CG: 11.11 ± 3.60 IG: 12.79 ± 5.51	< 0.001	0.214	0.448	0.010	0.158	0.036
**Gait speed (sec)**	CG: 0.93 ± 0.26 IG: 0.87 ± 0.20	CG: 1.08 ± 0.31 IG: 1.00 ± 0.23	< 0.001	0.409	0.713	0.002	0.274	0.021
**Reserve capacity (sec)**	CG: 0.25 ± 0.17 IG: 0.22 ± 0.12	CG: 0.29 ± 0.20 IG: 0.27 ± 0.16	0.054	0.065	0.806	0.001	0.525	0.007
**Gait speed dual-task (sec)** **IG** **:** ***n*** = 27 *	CG: 0.94 ± 0.27 IG: 0.83 ± 0.23	CG: 0.96 ± 0.28 IG: 0.90 ± 0.27	0.053	0.068	0.307	0.019	0.307	0.019

Results in SPPB Chair Rise showed a significant difference between IG and Chair Rise score, χ^2^ (3) = 8.857, *p* = 0.031

In SPPB Speed no significant between group allocation and Speed score was shown, χ^2^ (5) = 6.401, *p* = 0.269

The SPPB balance results showed no significant difference between group allocation and balance score, χ^2^ (5) = 6.949, *p* = 0.224

*Abbreviations*: *CG* control group, *IG* intervention group, *SPPB* Short Physical Performance Battery, *TUG* Timed “Up & Go”

^*^missing value due to concern about falling

The comparison of gait parameters showed a significant increase in gait speed in both groups after intervention. The interaction of group allocation and time was not significant. In the increase in reserve capacity, a trend over time became apparent. Performance at baseline and post intervention measurement and effects of the intervention are presented in Table 2.

### Effects of the intervention on gait parameters

Performance in gait parameters at baseline and post intervention measurement and effects of the intervention are presented in Table 3. Significant training-related improvements were obtained in both groups for gait speed, walk ratio, step length, step length max, stride length, stride length CV, stride length at maximum speed and stride length at maximum speed CV, double support and double support at maximum gait speed with large effect sizes.

**Table 3:** Effect of the intervention on gait variables

**GAITRite**®** variables**	**Baseline** **CG** **: ** ***n*** ** = 10** **IG** **: ** ***n*** ** = 7**	**Post intervention** **CG** **: ** ***n*** ** = 10** **IG** **: ** ***n*** ** = 7**	**Time**		Time*Group **allocation**		**Group** **allocation**	
			*p*-value	η^2^	*p*-value	η^2^	*p*-value	η^2^
**Gait speed** (m/s)	CG: 0.84 ± 0.18 IG: 0.80 ± 0.26	CG: 0.90 ± 0.18 IG: 0.97 ± 0.23	< 0.001	0.553	0.081	0.189	0.859	0.002
**Reserve capacity** (m/s)	CG: 0.33 ± 0.15 IG: 0.33 ± 0.15	CG: 0.34 ± 0.13 IG: 0.35 ± 0.19	0.690	0.011	0.919	0.001	0.895	0.001
**Gait speed dual task** (m/s)	CG: 0.83 ± 0.23 IG: 0.68 ± 0.26	CG: 0.81 ± 0.19 IG: 0.76 ± 0.32	0.579	0.021	0.351	0.058	0.366	0.055
**Walk ratio** mm/(steps/min)	CG: 4.90 ± 0.89 IG: 4.43 ± 0.83	CG: 5.11 ± 1.04 IG: 4.86 ± 0.54	0.011	0.363	0.300	0.071	0.402	0.047
**Walk ratio max** mm/(steps/min)	CG: 4.85 ± 1.29 IG: 4.39 ± 0.92	CG: 4.83 ± 1.28 IG: 4.96 ± 0.60	0.119	0.154	0.091	0.179	0.752	0.007
**Walk ratio dual task** mm/(steps/min)	CG: 4.95 ± 1.15 IG: 4.89 ± 1.59	CG: 5.14 ± 1.30 IG: 4.89 ± 1.66	0.729	0.008	0.743	0.007	0.808	0.004
**Step length** (cm)	CG: 49.37 ± 9.42 IG: 45.87 ± 10.29	CG: 52.26 ± 9.48 IG: 52.81 ± 7.26	< 0.001	0.559	0.093	0.177	0.742	0.007
**Step length max** (cm)	CG: 58.00 ± 11.21 IG: 53.51 ± 9.12	CG: 59.46 ± 10.67 IG: 62.01 ± 7.36	0.010**	0.369	0.054	0.226	0.835	0.003
**Step length dual task** (cm)	CG: 49.21 ± 10.60 IG: 43.92 ± 12.46	CG: 49.43 ± 9.19 IG: 51.19 ± 7.97	0.136	0.142	0.159	0.127	0.693	0.011
**Stride length** (cm)	CG: 99.23 ± 18.75 IG: 92.28 ± 20.69	CG: 104.95 ± 18.97 IG: 105.96 ± 14.61	< 0.001	0.548	0.100	0.170	0.741	0.008
**Stride length CV** (%)	CG: 4.54 ± 1.84 IG: 4.23 ± 1.80	CG: 3.54 ± 1.83 IG: 2.82 ± 1.83	0.037*	0.258	0.713	0.009	0.492	0.032
**Stride length max** (cm)	CG: 116.42 ± 22.51 IG: 107.73 ± 18.54	CG: 118.98 ± 20.71 IG: 124.17 ± 14.67	0.014*	0.340	0.060	0.216	0.851	0.002
**Stride length max CV** (%)	CG: 4.13 ± 2.09 IG: 5.31 ± 2.27	CG: 2.99 ± 1.78 IG: 2.44 ± 0.71	< 0.001	0.528	0.097	0.173	0.682	0.012
**Stride length dual task** (cm)	CG: 98.79 ± 21.03 IG: 88.38 ± 25.41	CG: 99.00 ± 18.50 IG: 102.67 ± 16.82	0.138	0.141	0.149	0.134	0.713	0.009
**Stride length dual task CV** (%)	CG: 8.44 ± 7.35 IG: 9.38 ± 6.18	CG: 6.18 ± 6.06 IG: 7.17 ± 4.90	0.242	0.090	0.991	< 0.001	0.706	0.010
**Double support** (sec)	CG: 0.37 ± 0.06 IG: 0.40 ± 0.10	CG: 0.35 ± 0.07 IG: 0.34 ± 0.10	0.009	0.374	0.228	0.095	0.785	0.005
**Double support max** (sec)	CG: 0.26 ± 0.06 IG: 0.29 ± 0.10	CG: 0.25 ± 0.05 IG: 0.24 ± 0.10	0.003	0.450	0.078	0.192	0.812	0.004
**Double support dual task** (sec)	CG: 0.42 ± 0.24 IG: 0.49 ± 0.14	CG: 0.41 ± 0.14 IG: 0.73 ± 0.83	0.406	0.047	0.329	0.064	0.226	0.096

*Abbreviations*: *CG* control group, *IG* intervention group, *CV* Coefficient of Variation

The analysis of the interaction of group allocation and time did not show any significant difference. In this analysis, a trend was found for the parameters step length at maximum gait speed and stride length at maximum gait speed in favour of the IG.

## Discussion

In this study we examined SSE in the context of inpatient early geriatric rehabilitation in comparison to conventional physiotherapy. The main outcome was the improvement of the physical function. The early geriatric rehabilitation program with replacement of three cPT sessions per week by SSE is as effective as the usual rehabilitation program, measured with the SPPB und TUG. The results support the assumption that SSE is also effective over a comparatively short training period [19–21]. This study showed that SSE improves physical function of the lower extremity also in a population of frail older adults requiring geriatric acute care. This is probably due to the comparatively high training intensity that SSE entails. During the training session, the participant stood and walked throughout. This resulted in a higher number of steps, which additionally strengthens the lower extremity. cPT, on the other hand, often took place in a seated position or involved only short distances from the patient's room to the treatment room. Since this was the first investigation in this setting, further research is needed to confirm this assumption. This could be achieved, for example, by sensor-based recording of the number of steps. The significant improvement in the chair rise score in favor of the intervention group suggests an improvement in lower extremity muscle strength. Previous studies have shown a correlation of lower extremity muscle strength with standing time, gait speed, and number of steps [34].

Both groups improved their performance in the gait parameters equally over time. However, the analysis of the interaction of group allocation and time of gait parameters showed a trend for more improvement in step length at maximum speed and stride length at maximum speed in the SSE group. To our knowledge, there are currently no studies that have specifically investigated the effect of SSE on gait parameters. There are also no studies of step-based exercises that would be comparable to this study's target population. Therefore, these results cannot be embedded in the research landscape and should be further investigated.

The implementation of the study proceeded without any falls or other adverse events. Therefore, it can be assumed that SSE can be safely implemented in early geriatric rehabilitation. Dropout from the study due to the training occurred in only one case, showing excellent adherence. This is consistent with previous study results [11, 16]. It is important to note that an intention-to-treat (ITT) analysis was not conducted. Although there were only two datasets that could have been included in this analysis. In general, a missed ITT analysis can lead to an overestimation of effects. Therefore, an ITT analysis should be considered in future studies. The playful aspect of SSE training offers the opportunity to increase participants’ motivation and promote adherence. Physiotherapists who carried out the SSE training in this pilot study reported that the training had added value through joyful moments with the participants and active interaction with them. SSE offers an extension of therapeutic methods in the rehabilitation process. A wider range of available methods allows for better addressing of individual patient preferences and promotion of adherence. The clientele included in this study is described via the reported Barthel Index. When discussing the implementation of SSE in geriatric early rehabilitation, it is important to consider that SSE is not a training program suitable for every patient.Importantly, the program is effective over a relatively short period of time. As the training program can be implemented at low cost it is a playful additional component for cPT. SSE was originally designed as a group training program. Therefore, studies on the effects of SSE have been conducted in a group setting so far. To the best of the authors' knowledge, SSE has not yet been studied as an individual training method in geriatric early rehabilitation. SSE proved to be feasible and effective in combination with cPT in individual training in early rehabilitation in geriatric inpatients in this pilot study. The advantage of group training could be the social interaction of the participants. Previous studies suggest that group interaction has a positive impact on motivation [20]. Also, group training would be more economical in terms of time and cost, since only one trainer is needed [11, 20]. A disadvantage could be the lower training intensity. Within the training duration, the individual participant would walk fewer step patterns. This would reduce the number of steps and possibly strengthen the lower extremity less. For this purpose, further research is needed to compare individual training with group training in this specific setting. By using sensor-based measuring instruments, the participants' activity during the training session could be precisely recorded and evaluated.

Compared to other studies on the effect of SSE on gait characteristics and physical function, the training duration of this study was short [36]. This is due to the special setting and should be considered as a limitation. Also due to the setting, no exclusive SSE training could be investigated. The measurement results refer to cPT in combination with SSE compared to cPT. No statement can be made about the frequency with which SSE training is effective. To our knowledge, no data are available on the frequency of SSE training and the resulting benefit.

When interpreting the results, it must be considered that the Chair Rise Test was given as a score. In order to obtain more meaningful results, the seconds required should also be recorded as test performance in further studies.

A further limitation was that we did not prioritize in the motor-cognitive test situation for the participants on either gait or cognitive task. In addition, we did not obtain the motor cognitive inference between the two conditions.

Another limitation is the small sample size in the GAITRite® measurement. However, despite the small sample size, effects over time could be shown for both groups with large effect sizes and trends in favor of the IG for some gait characteristics. Thus, these results can be used as a starting point for further research.

## Conclusions

As measured by the SPPB, all participants improved clinically meaningful over the rehabilitation period independent of group allocation [35]. Despite the relatively short training period, substantial effects of three SSE sessions per week replacing cPT on physical function and gait were shown during early geriatric rehabilitation. SSE in this alternation with cPT proved to be as effective as cPT alone. Thus, SSE is a playful additional component for cPT.

Because of the high cognitive demands of the training, the cognitive performance of the participants should also be examined in this specific setting.

Another aspect for further research is the evaluation of the participants' activity during training. This could be represented by sensor-based measurement of the actual number of steps in SSE units compared to cPT units.

After SSE proved to be feasible and effective in individual training in early rehabilitation in geriatric inpatients who have participated in the entire program with at least 6 SSE units, a group training could also be investigated in this setting.

### Supplementary Information

**Supplementary Materials 1.**

## Data Availability

The data that support the findings of this study are available on reasonable request from the corresponding author.

## Abbreviations

CG: Control Group
cPT: Conventional Physiotherapy
EQ-5D: EuroQol- 5 Dimension
FES-I: Falls Efficacy Scale – international
IG: Intervention Group.
ITT: Intention-to-treat
MMSE: Mini Mental Status Examination
RCT: Randomized controlled trial
SPPB: Short Physical Performance Battery
SSE: Square-Stepping Exercise
TUG: Timed „Up & Go
