# Supplementary material for: Square-stepping exercise in older inpatients in early geriatric rehabilitation. A randomized controlled pilot study
Source: BMC Geriatr. 2024 Apr 10;24:326. doi: 10.1186/s12877-024-04932-3 (PMC11005258; doi:10.1186/s12877-024-04932-3)
Supplement: Supplementary file 1 — Supplementary Materials 1. [file 12877_2024_4932_MOESM1_ESM.docx]

**Table S1.** Evaluation of differences in baseline characteristics of participants and dropouts

| **Characteristic** | **CG**  **(n=29)** | **CG drop-out/exclusion**  **(n=7)** | **p-value** | **IG**  **(n=29)** | **IG drop-out/exclusion**  **(n=7)** | **p-value** |
| --- | --- | --- | --- | --- | --- | --- |
| Age Mean ± SD, (min-max) | 78.9 ± 6.73  (65-88) | 79.1 ± 8.46  (67-94) | 0,904 | 79.4 ± 7.08  (63-90) | 77.6 ± 9.19  (62-89) | 0,670 |
| Woman (number) | 13 | 5 |  | 14 | 4 |  |
| Education (years) Mean ± SD, (min-max) | 11.5 ± 1.97  (7-16) | 10.6 ± 1.18  (8-12) | 0,114 | 12.2 ± 3.10  (7-20) | 11.7 ± 3.28  (8-17) | 0,904 |
| Mini mental status examination (score) Mean ± SD, (min-max) | 27.9 ± 1.63  (23-30) | 28.9 ± 0.83  (28-30) | 0,207 | 27.5 ± 1.95  (23-30) | 27.7 ± 1.83  (25-30) | 0,764 |
| Barthel Index (score) Mean ± SD, (min-max) | 61.9 ± 12.06  (40-85) | 57.9 ± 14.6  (45-85) | 0,238 | 64.5 ± 13.32  (40-90) | 58.6 ± 14.57  (40-85) | 0,379 |
| SPPB (score) Mean ± SD, (min-max) | 7.03 ± 2.93  (1-12) | 4.6 ± 1.4  (3-7) | 0,013 | 6.62 ± 2.24  (4-11) | 5.6 ± 1.99  (3-8) | 0,271 |
| TUG (sec) Mean ± SD, (min-max) | 14.64 ± 6.65  (5-32) | 19 ± 5.55  (12-28) | 0,061 | 18.07 ± 12.88  (7-78) | 15.8 ± 4.57  (8-22) | 0,726 |
| Gait speed (m/sec) Mean ± SD, (min-max) | 0.93 ± 0.26  (0.5-1.43) | 0.76 ± 0.18  (0.44-0.98) | 0,873 | 0.87 ± 0.20  (0.38-1.25) | 0.86 ± 0.18  (0.62-1.24) | 0,928 |
| Maximum gait speed (m/sec) Mean ± SD, (min-max) | 1.19 ± 0.35  (0.73-1.85) | 1.12 ± 0.31  (0.62-1.54) | 0,711 | 1.09 ± 0.27  (0.56-1.64) | 0.98 ± 0.23  (0.48-1.19) | 0,920 |
| FES-I (score) Mean ± SD, (min-max) | 23.7 ± 6.98  (16-41) | 32.7 ± 11.83  (19-45) | 0,043 | 23.3 ± 7.54  (16-52) | 23.1 ± 3.72  (16-28) | 0,496 |
| EQ-5D Level of health (%) Mean ± SD,  (min-max) | 62.5 ± 20.82  (20-100) | 57.9 ± 15.55  (30-80) | 0,631 | 60.2 ± 21.19  (20-90) | 56.4 ± 10.25  (40-75) | 0,509 |
